# Illness Narratives Without the Illness: Biomedical HIV Prevention Narratives from East Africa

**DOI:** 10.1007/s10912-024-09862-0

**Published:** 2024-06-26

**Authors:** Jason Johnson-Peretz, Fredrick Atwine, Moses R. Kamya, James Ayieko, Maya L. Petersen, Diane V. Havlir, Carol S. Camlin

**Affiliations:** 1 Obstetrics, Gynecology & Reproductive Sciences, University of California, San Francisco (UCSF), San Francisco, CA, USA; 2 Infectious Diseases Research Collaboration, Kampala, Uganda; 3 Kenya Medical Research Institute (KEMRI), Kisumu, Kenya; 4 Department of Medicine, Makerere University College of Health Sciences, Kampala, Uganda; 5 Biostatistics, Epidemiology, and Computational Precision Health, University of California, Berkeley, Berkeley, CA, USA; 6 Division of HIV, Infectious Diseases, and Global Medicine, University of California, San Francisco (UCSF), UCSF Box 0874, San Francisco, CA 94143-0874, USA

**Keywords:** Illness narratives, Prevention, East Africa, Luo, Swahili, HIV

## Abstract

Illness narratives invite practitioners to understand how biomedical and traditional health information is incorporated, integrated, or otherwise internalized into a patient’s own sense of self and social identity. Such narratives also reveal cultural values, underlying patterns in society, and the overall life context of the narrator. Most illness narratives have been examined from the perspective of European-derived genres and literary theory, even though theorists from other parts of the globe have developed locally relevant literary theories. Further, illness narratives typically examine only the experience of illness through acute or chronic suffering (and potential recovery). The advent of biomedical disease prevention methods like post- and pre-exposure prophylaxis (PEP and PrEP) for HIV, which require daily pill consumption or regular injections, complicates the notion of an illness narrative by including illness prevention in narrative accounts. This paper has two aims. First, we aim to rectify the Eurocentrism of existing illness narrative theory by incorporating insights from African literary theorists; second, we complicate the category by examining prevention narratives as a subset of illness narratives. We do this by investigating several narratives of HIV prevention from informants enrolled in an HIV prevention trial in Kenya and Uganda in 2022.

## Background

“For, from a word, a group of words, a sentence and even a name in any African language, one can glean the social norms, attitudes and values of a people.” — Nigerian poet Gabriel Okara (Okara 1963; Ngũgĩ 1992, 8)

Healthcare messaging which resonates with the values and culture of a population is especially important for ongoing health literacy, especially in contexts of novel disease prevention technologies (mRNA vaccines, oral and injectable PrEP, PEP) and in the face of competing values affecting community and family health. Personal narratives are one means by which the links between body, self, and society can be articulated. In this paper, we propose *illness prevention* narratives — narratives about adopting biomedical disease prevention methods — as a sub-genre of the larger category of illness narratives ripe for analysis. We focus specifically on narratives around HIV prevention in East Africa, a geographic region with consistently high HIV transmission rates, especially among youth. Since “language carries culture, and culture carries, particularly through orature and literature, the entire body of values by which we come to perceive ourselves and our place in the world” (Ngũgĩ 1992, 16), we further bring contemporary African literary theory to bear on the examination of those narratives in an African context, providing both a corrective to an over-emphasis on Euro-American narrative theories of illness and an opportunity to present narratives already enculturated in the region where we work.

Illness narratives are first-person accounts of how the narrator experiences a sense of ill-being. Narratives, as distinguished from stories, are templates tailored to individual circumstances through stories, and they help people make sense of illness in both personally and socially meaningful ways (Frank 2009). For social scientists, illness narratives provide insight into how patients understand the why and how of illness causation and treatment while simultaneously illuminating the broader social and structural contexts of patients, their communities, and the clinicians which the illness process links together (Hydén 1997; Bury 2001; Riessman 1990; Shapiro 2011; Williams 1997). In this way, illness narratives are not only forms of meaning-making but also resources for health education and culturally sensitive messaging (Le, Miller, and McMullin 2017; Kumagai 2008).

Illness narratives, along with statistics and epidemiological reports, have documented how the introduction of antiretroviral medications (ARVs) and the rollout of universal test and treat protocols, have changed the lives of people with HIV. Susan Reynolds Whyte and her team in Uganda, for example, recorded illness narratives on the cusp of HIV moving from a terminal diagnosis to a chronic, managed disease, thanks to the widespread introduction of affordable antiretroviral therapy in the early 2000s (Whyte 2015). Progress in ending the HIV epidemic has also continued to gain momentum through the introduction of biomedical prevention therapies.

In 2012, the WHO recommended HIV-negative individuals use ARVs to prevent infection by HIV in case of exposure (WHO 2012). This preventive treatment is called pre- and post-exposure prophylaxis, or more commonly, PrEP and PEP. Importantly, introducing PrEP and PEP to *prevent* HIV infection adds a layer of complexity to the category of illness narratives, since people use these biomedical tools precisely to prevent illness. How can one narrate the experience of an illness that never comes about? Yet people do relate accounts of why they choose to take PrEP and what it means to them. So perhaps a better question is, *do illness prevention narratives exist as a subset of illness narratives?*

To answer that question, we briefly review the historical development of illness narrative studies to illustrate key components of the theory, before presenting narratives from our participants about their choice to engage with biomedical HIV prevention methods during a test-and-treat trial in rural Kenya and Uganda. Because illness narrative studies were originally based on Euro-centric models of literary criticism, we introduce elements drawn from African literary theorists to develop existing theory and adapt it to an East African context. Importantly, the development of mid-twentieth century European literary criticism coincided with the period of independence for many African nations, and African writers of English, French, and Portuguese of the time discussed those theories in terms relating to their contexts within post-colonial Africa (Ngũgĩ 1992, 4–9). Together, the theoretical enrichment provided by an African literary lens and the empirical evidence from field sites in East Africa help provide answers to our research question.

### Illness narratives: a brief introduction

Illness narratives are inevitably entangled in the politics of language, which has a bearing on the self in society. As the Kenyan literary theorist Ngũgĩ wa Thiong’o writes, “The choice of language and the use to which language is put is central to a people’s definition of themselves in relation to their natural and social environment, indeed in relation to the entire universe” (Ngũgĩ 1992, 4). Discrepancies between professional, personal, and social understandings of ill-being, as distinguishable through the terms *disease, illness*, and *sickness*, highlight the cultural understandings, social networks, relational and agentic constraints, and personal values at play in these experiences, especially as they touch on “the constitution of the self” in society (Kelly and Dickinson 1997; Sakalys 2000; Marinker 1975; Young 1982). In this set, *disease* is generally taken to be biomedically or provider-defined ill-being, using categories appropriate to medical professionals. This may include physician accounts of the progress of the patient experiencing that disease, as in case studies. *Illness* — from which the genre of ‘illness narratives’ takes its name — is the personally felt and interpreted experience of ill-being, whether that experience falls within medically defined categories of disease or not. Finally, *sickness* is the socially deployed and socially understood state of ill-being, recognized as something which alters one’s social positioning and relationships (Good 1993; Parsons 1975, 2013 [1951]).

The distinction of an illness narrative from a case study turns on the answer to the question, *Who tells the illness narrative?* (Bury 2001; Riessman 1990). As Kleinman defines it, “the illness narrative is a story the patient tells, and significant others retell, to give coherence to the distinctive events and long-term course of suffering… The personal narrative does not merely reflect illness experience, but rather it contributes to the experience of symptoms and suffering” (Kleinman 2020). Illness narratives move beyond ‘pathographies’ which focus merely on a diseased organism to encompass social relations and contexts while positioning the self-experiencing-illness, rather than the disease, at the center (Wiltshire 2000; Hydén 1997).

As one aspect of the *narrative constitution of the self*, illness narratives position the narrator on a particular moral and social field of values and relationships (Goffman 2002 [1959]). As Ngũgĩ notes, “Values are the basis of a people’s identity, their sense of particularity as members of the human race. All this is carried by language;” and the ‘self’ is an autobiographical narrative at its heart (Ngũgĩ 1992, 15). Kelly and Dickenson identify four discernible components in such narratives: an account with evaluative relations between events in time; a cosmology which presents assumptions about causal connexions between events and impersonal moral purposes; an observation of power relationships such as social structures which enable or constrain agency with respect to others; and finally, a concept or concepts of self as an object of continuity and change. These elements “provide the basis for practical inter-subjectivity” (Kelly and Dickinson 1997; Schutz and Wagner 1970). In this sense, illness narratives are always edited (some elements deemed irrelevant are left out of the telling) and strategic (deployed to gain the approbation of a particular audience or interlocutor or to mobilize resources from the social field) (Riessman 1990; Bury 2001).

Such narratives can be developed and deployed in social settings where individuals find meaning as part of a group sharing the same diagnosis, the same medication, the same challenges, or the same routine. This sort of ‘biosocial identity’ is often a feature of illness narratives when the narrator participates in patient groups (Nayar 2021). When deployed as part of an education or policy campaign, these illness narratives also serve social narrative functions ultimately as part of a discourse of health and wellness (Wong and King 2008; Sharf and Vanderford 2003).

While research initially subordinated illness narratives to studies of identity and self, illness narrative research in the 1980s placed biomedical efforts around the management of disease in tension with treatment approaches which aimed to cure the disease. Doing so allowed social scientists to critique the dominance of reductionistic biomedical theories of sickness characteristic of the 1980s and earlier, which tended to see patients less as people and more as case studies of a particular disease (Bury 2001; Gerhardt 1989; Hydén 1997; Shapiro 2011; Frank 1994; Hawkins 1990). As the decade wore on, the utility of illness narratives in the politics of patient-centered care became increasingly obvious, especially as seen through the lenses of AIDS, disability, and policy change in the United States (Hydén 1997; Sakalys 2000; Ezzy 2000). In other words, through illness narratives, doctors began to pay increasing attention to how the person suffering the illness or disease is a key player not only in articulating the experience of suffering, but also for co-creating the therapeutic agenda to ease that suffering.

### Illness narratives: theory, types, genre

#### Theory

Illness narratives share several key elements. First, illness narratives typically contain a disruptive event — the illness — usually accompanied by suffering and a change in daily life. This disruption is then often followed with a narrative reconciliation either towards chronicity or cure. As Hydén puts it, “The patient seeks to find an explanation for and to understand his or her illness in order to find an ending for the illness narrative” (Hydén 1997; Hawkins 1990). The narrative thus tends to be organized around time and its consequences (Bury 2001; Riessman 1990). Despite the constant presence of time in such narratives, the narrative resolution can be quite open-ended (Ezzy 2000). Good et al. (1994) refer to this as the ‘subjunctive mode,’ “open to mystery, potency and change.” This openness not only gives space for hope and justification for ongoing care-seeking, but also recognizes the ‘incompleteness’ of the illness — or in our cases, prevention — experience: endings can be only hypothetical or hoped for (Good et al. 1994). Whatever ending(s) is presented, whether actual or hypothetical, orients the whole and allows not only for relating the constituent elements but also for identifying what is most meaningful in the experience. Without an end, the narratives remain ambiguous and search for either a meaning or resolution, a way of situating the past to make sense of the present (Good et al. 1994). This leads into the second and third common features of illness narratives already mentioned: their edited and strategic construction and deployment (Young 1982; Riessman 1990; Shapiro 2011), and their ongoing reconstitution of the self as an integrated whole, done in both instances for an audience and specific context (Hydén 1997).

The European orientation to time, however, has been criticized by several African literary theorists including Tanzanian author Kezilahabi and the Kenyan philosopher-priest John Mbiti. For these two authors, the African concept of time is cyclical and the *present* is the standard of being and becoming against which an ideal scenario is measured; the ideal generally does not exist in the future (Lanfranchi 2012; Kezilahabi 1985). A secular European or North American might argue the future (and the past) are indeed considered in African contexts by reference to the ancestors and yet-to-be-born descendants; but in African contexts, the ancestors and the yet-to-be-born are already-present figures, much as they are in South American magical realism and the Roman Catholic and Byzantine Orthodox communion of saints (Adade-Yeboah 2016). Further, as the Cameroonian Jesuit Jean-Luc Enyegue noted when examining the work of the Tanzanian theologian Laurenti Magesa, in Africa “time is a return to the past with the ultimate aim of creating a more humane and life-giving human person and society” in the present (Magesa 2014; Enyegue 2021; Mugambi et al. 2022). Thus, in an African illness (prevention) narrative, the orientation of the story should not be expected to project outwards into the future of oneself, but rather outwards into present relations with others. Nevertheless, in a world of increasing hybridity between global and local, African, South Asian, and European, this cosmological framework may be changing as the siloes which reserved certain languages for expressing certain thoughts and not others begin to interpenetrate one another (Ngũgĩ 1992, 96–104; Appiah 2017; Miano 2020; Sankara 2002).

#### Types

Schematically apart from genre is the closely allied concept of ‘type.’ Bury usefully categorizes illness narratives into three types: contingent, moral, and core narratives. Within this scheme, the typologies of other researchers can be situated (Bury 2001). *Contingent narratives* address beliefs about the origins of disease, the proximate causes of an illness episode, and the immediate effects of illness on everyday life.

*Moral narratives* provide accounts of, and help to constitute, changes between the person, the illness and social identity. This type of narrative helps to (re-)establish the moral status of the individual. It may also help maintain social closeness by ensuring the illness not become a social wedge between the narrator and his or her intimates. It is akin to what Ngũgĩ calls “the search for a liberating perspective within which to see ourselves clearly in relationship to ourselves and to other selves in the universe. I shall call this ‘a quest for relevance’…” (Ngũgĩ 1992, 87). It is also the scene in which illness can become a symbol or metaphor, participating in larger moral narratives reflecting the effects of illness on the body — tuberculosis a metaphor for spiritualization of the person (in European opera, for example) or cancer for something like resentment, envy, or grief eating away at them (Riessman 1990; Sontag 2001; Kleinman 1997).

Finally, *core narratives* reveal connections between the layperson’s experiences and deeper cultural levels of meaning attached to suffering and illness (Le, Miller, and McMullin 2017; Kelly and Dickinson 1997). Core narratives, which touch closely upon the self and identity, have sub-genres of heroic, regressive/progressive, tragic, ironic, comic, and quest narratives (Bury 2001). In an African context, tragic narrative often turns on the conflict between an individual rooted in tradition and a community which embraces social change (Gromov 2019). The importance of a tragic type illness (prevention) narrative in Africa would then lie in the light they shed on links between disrupted experience and agency, as well as on identity and cultural continuity.

Illness narratives can be characterized as stable, progressive, or regressive, depending on whether a chronic disease is moving towards resolution, towards death, or maintained simply as ‘how things are.’ This is similar to Ezzy’s linear restitutive, linear chaotic, and polyphonic narratives, wherein the narrator is oriented towards future plans, a lost potential, or the uncertainties and appreciation of the present (Ezzy 2000; Kelly and Dickinson 1997; Bury 2001; Mancuso et al. 1983; Robinson 1990). In contexts where the attitude towards time is less linear and more fluid, a narrative’s temporal orientation may not be as clear cut as in narratives from the global North or West. Especially in the ‘new’ Swahili novels, quests are often left open-ended, and their resolution left for the reader — who adopts the moral of the tale — to perform (Gromov 2019).

#### Genre

With roots in folklore and narrative studies more generally, several authors argue that illness narratives often conform to the patterns of existing literary genres (Kelly and Dickinson 1997; Bury 2001; Hydén 1997). Thus, illness narratives can be broadly classed as tragic, in which the narrator starts well and ends in misfortune; or comic, in which things look bad at the beginning but end up well by the end. Another example would be the hero’s journey, in which the person with an illness is the protagonist and the illness (or medical system) is the antagonist or challenge the hero must overcome to return back to the self. As in Joseph Campbell’s ‘monomyth,’ the story usually has an ‘arc,’ with a beginning, a crisis which precipitates a plot movement to the middle set of challenges, and finally a satisfying, though not always ‘happy’ end (Kezilahabi 1985; Segal 2021; Campbell 2008). Illness narratives also frequently end with an ultimate moral or meaning derived from the story and given to others for their edification, though sometimes this meaning is particular and revealing of the patient’s own sense of being-in-the-world (Hydén 1997; Frank 1997; Shapiro 2011; Riessman 1990). In this respect, they often function as secular versions of ‘conversion narratives’ (Hawkins 1990).

Importantly, these genres are themselves culturally informed. Given the polyphony of language, historical experience, and cultures throughout the African continent, an overarching ‘African’ narrative may be difficult to come by. Nevertheless, Ngũgĩ wa Thiong’o confronted not only the question of what counts as ‘African literature,’ but in dialogue with many other authors on the African continent sought particularly African approaches to such literary forms as theatre, epic, and fiction (Ngũgĩ 1992). Western models, he points out, may not match contemporary African models. We argue that this applies both in literature and in illness (prevention) narratives. We have already mentioned the issue of time, but the mismatch extends as well into African concepts of the hero (Kezilahabi 1985). Focusing specifically on the Tragic Hero in African Literature, African heroes have tragic flaws; in literature set in colonial and post-colonial African nations, these flaws often involve getting caught between upholding tradition against one’s community and embracing communally sanctioned cultural change (Adade-Yeboah 2016). As the Acholi poet Okot p’Bitek cautions while agreeing with development, “Don’t desert your ancient culture: ancient cultures are not for desertions; cultures are not for abandoning” (lo Liyong 1993). Importantly, in African novels, tragedy often functions as a regenerative force for the community presented in the story (Adade-Yeboah 2016).

Many African novels today draw on and blend elements from multiple genres, including oral (epic) literature, folklore, and poetry, to create new, uniquely African genres. In particular, the quest theme is making a renewed appearance in the plots of the ‘new’ Swahili novel (Gromov 2019). In these often dystopian settings, the hero is an ordinary person, not endowed with great magic or lineage, in contrast to earlier epic tales (Okpewho 1981). Their task is often to create the future world, and find a way out of the predicaments created by colonialism, globalism, corruption, individualism, and ill-used power (Gromov 2019). Swahili quest narratives are similar to other quests, whose narratives focus on a person’s calling, and how to be worthy of one’s life and its events. (Frank 2009). In this, the plot device resembles other quest narratives, but with local inflexions which parallel the conflicts faced by African tragic heroes. Importantly, the results of the quest are not always resolved; while not necessarily admitting an explicit cyclicality (however optimistic), the novels still espouse more encompassing views which do not admit of easy or complete resolutions. One might therefore plausibly expect these themes, including an open-ended resolution, to appear in illness prevention narratives.

### Illness narratives: applications

Illness narratives participate in the creation of cultures of health, morality, suffering, community, and politics, at times reinforcing cultural norms and at times changing them. Ngũgĩ argues that,

Communication between human beings is also the basis and process of evolving culture. In doing similar kinds of things and actions over and over again under similar circumstances, similar even in their mutability, certain patterns, moves, rhythms, habits, attitudes, experiences and knowledge emerge. Those experiences are handed over to the next generation and become the inherited basis for their further actions on nature and on themselves. (Ngũgĩ 1992, 14)

Illness narratives, as communicative by nature, are not exempt from this process of cultural creation. Hydén cites five uses for illness narratives, which bear on culture-creation (Hydén 1997). The first is to transform illness events into some meaningful whole; the second is to reconstruct life history in light of illness; while the third is to explain, understand, or relate to the illness in some fashion (especially in finding the cultural or moral causes of it). Drawing on the third use, medical education began to incorporate illness narratives for their pedagogical value in sensitizing medical students to culturally diverse understandings of ill-being and treatment (Fadiman 2012; Kumagai 2008; Sakalys 2000). Though this has sometimes come with drawbacks, the utility of illness narratives for uncovering cultural differences in cosmology and identity has been useful in tailoring messaging and health literacy that moves with, rather than against, the grain of local patient understandings of ill-being (Le, Miller, and McMullin 2017; Shapiro 2011). They further teach both empathy and instances of conscious and unconscious bias which mitigate against empathy on the part of providers towards minority patients (Ross, Lypson, and Kumagai 2012; DasGupta and Charon 2004).

A fourth use for an illness narrative is through a strategic interaction which projects an identity or achieves certain ends. This sometimes broadens out to the fifth use which transforms the illness from an individual into a collective phenomenon, as happened with the disability and AIDS political movements of the late 1980s in the United States. In these instances, illness narratives were mobilized in political battles to gain political protections, free up government funding for a cure (or treatment), and in general recognize that those with disabilities or HIV were in fact persons — “people living with HIV” (Crimp 2004; Takemoto 2003; Diedrich 2022; Luce 2013; Fox 1992).

With respect to the fourth and fifth uses, researchers discovered illness narratives seem to have a therapeutic value beyond merely communicating a sick role. In some cases, such narratives help co-create a ‘therapeutic agenda’ in which both the medical provider and the patient could participate (Le, Miller, and McMullin 2017; Sakalys 2003). Thus, one theoretical question — whether illness narratives heal — was answered by psychologists who applied theory to their own practice. Victor Frankl’s ‘logotherapy’ is a type of narrative or discursive deployment aiming to identify individual purposes or projects which give meaning to a person’s life (Frankl 1967, 2014). Partly drawing from logotherapy, positive psychologists later used narrative therapy to improve health outcomes by resituating the patient’s self-perception in society and integrating past harmful experiences in ways which permitted the patient to engage with renewed purpose, joy, amusement, equanimity, or interest in life after distressing events (Achor 2018; Seligman 2002; Csikszentmihalyi and Rathunde 1993; Gable and Haidt 2005).

### Illness prevention narratives: some questions

With the above introduction in place, we can now revisit the initial question about how the current introduction of biomedical prevention for HIV complicates the category and theory of illness narratives, turning an *illness narrative* into an *illness prevention narrative*. If illness is a sense of ill-being, how does prevention fit in?

We argue that a sense of risk can be included in a sense of ill-being, in the same way that a sense of security helps constitute and define well-being (WHO 1989). Similar to a sense of illness leading one to seek care, so also a sense of risk prompts one to seek ways to mitigate that perceived risk when possible (Price et al. 2018; Hill et al. 2020). This can extend beyond the realm of infectious disease like HIV to include the choice to undergo (or to refuse) prenatal and pre-disease genetic testing (Timmermans and Buchbinder 2010; Hunt, Castañeda, and De Voogd 2006). We argue that preventive medications are also one form of care seeking, illustrative of a sense of risk and search for security. Conversely, it seems to us, one can reject a preventive method and the narrative which goes along with it in order to maintain a different narrative identity of health, resistance, or strength, again, not unlike those who reject pre-natal testing procedures. Five questions will therefore guide the discussion and analysis of the prevention narratives we present below.

First, *what sort of narrative is this?* If suffering is not necessarily tied to an active illness, nor a pathography the defining mark of an illness narrative, what do these narratives disclose? Second, *What disruption does it cover?* Does a disruption exist, and if so, of what sort? Third, *What moral purpose does it serve?* To what extent does it constitute the self, and what self is that, exactly? Fourth, *What therapeutic value can it have?* If it does not ‘heal’ a person — because the body is ‘saved’ through a biomedical intervention, what purpose does the narrative serve? Is therapeutic value necessary for an illness narrative? Finally, *Can illness prevention theories further illness narrative theories in general?* What is the limit or constraint in existing illness narrative theories? What new light can a theory of illness prevention narratives shed on social processes and social ties?

Answering these questions will not only give researchers insight into discourses around health and illness, but also help those of us engaged in public health address concerns raised by members of the communities where we live and work in culturally specific and appropriate ways through messaging which acknowledges the challenge of competing values, perspectives, and personalities and resonates with the life experiences of our audiences. In this way, we can appropriately encourage people to seek preventive care when warranted and create cultures of health within our communities.

## Methods

The narratives of this article are drawn from in-depth, semi-structured interviews with participants from the SEARCH-SAPPHIRE study in East Africa. The overall objective of SEARCH-SAPPHIRE Study (NCT04810650) is to determine how to reduce HIV incidence and deaths and improve overall health using a community precision health approach that incorporates new products and patient-centered, multi-disease delivery. The study is being conducted in 16 communities (~ 10,000 persons/community) in rural Kenya and Uganda. In the first phase, the study piloted (a) dynamic choice treatment — an intervention to support mobile persons with HIV in their treatment program; and (b) dynamic choice HIV prevention model with participants from ante-natal clinics (ANC), outpatient departments (OPD), and village health teams (VHT). These participants are offered a range of methods to prevent HIV, such as condoms, PrEP, PEP, and HIV self-test kits. They have the option to switch methods at will. Our narratives originated from this latter, prevention-focused population.

### Data collection and analysis

A gender-balanced team of trained qualitative researchers who were native speakers of the local languages administered 100 in-depth semi-structured interviews (ANC, *n* = 19; mobile populations, *n* = 18; OPD, *n* = 19; VHT, *n* = 18; provider, *n* = 26) from November 2021 through March 2022. Interviews explored life and community contexts, experiences with the intervention, preferred prevention or treatment methods, patient-provider interaction, and discussions with peers about HIV, HIV prevention, and HIV stigma. The first and senior authors (JJP, CSC) with input from the interviewing team (LO, AO, CA, FA, TOA) developed the interview guides, building on earlier interviews conducted in these communities on HIV, risk perceptions, stigma, and mobility. The team translated the guides into DhuLuo, Swahili, and Runyankole; interviews were conducted in those languages or English according to participant preference.

Researchers ensured confidentiality by conducting audio-recorded interviews in private and comfortable locations convenient for the participants such as clinic rooms, outside, or in homes, after consenting them verbally and in writing. The team then transcribed and translated the audio recordings into English. After translation and transcription of the interviews, a six-person team (LO, AO, CA, FA, TOA, JJP) coded them using a codebook developed from both *a priori* and inductive codes derived from line-by-line coding of a subset of interviews (Johnson-Peretz et al. 2024). The codebook included a code for provider interviews called ‘case studies,’ but during data collection and analysis, we encountered several self-contained narratives of patients taking up and accessing PrEP and PEP. We therefore expanded the code name and definition to ‘case studies and illness narratives.’ As a team, we discussed whether the term ‘illness narratives’ was appropriate to these stories, since they concerned the *prevention* of an illness, rather than the personal and social experience of living with an illness. This paper is an effort to answer that question.

Once the team finished coding all transcripts, we downloaded excerpts coded with the illness narratives tag, which yielded 104 excerpts, from all cohorts, including case studies and narratives concerning ARV treatment and adherence. From this set, we chose to analyse data only from participants in the ante-natal clinic (ANC), out-patient department (OPD), and village health team (VHT) cohorts (i.e., the dynamic choice prevention cohort). We excluded interviews with Mobile participants on HIV treatment since their illness narratives concern HIV treatment, rather than HIV prevention. We also excluded interviews with providers from the analysis here, as provider stories of participants come closer to the definition of ‘case studies’ than ‘illness narratives.’ (No providers gave first-hand accounts of their own prevention experiences.) We ultimately identified eleven narratives which could plausibly be considered illness prevention narratives. We then used a framework table to look for patterns in the narratives, taking Bury and Riessman’s method of comparing whole accounts across a cohort to identify the range of narrative forms in order to generate second-order models for theoretical development (Bury 2001; Riessman 1990). Further analysis eliminated four of the excerpts (one from a VHT; one pertained to seeing people die of HIV, but not directly tied to taking prevention meds; two were removed due to space constraints and being outliers. We include these in supplementary materials), and we present the seven remaining narratives below.

### Author(s) position statement(s)

The team-based analytical approach was enriched through a lens of lived experience by research team authors who had first-hand understanding of the social (and literary!) contexts in which these narratives were gathered (FA, CA, LO, AO, TOA). The research team members, who gathered data in the local languages Dholuo (LO, AO, TOA) and Runyankole (CA, FA), also engaged in codebook development and data analysis. This ensured that the translation and interpretation of the data respectfully captured the nuances of participants’ voices. The first authors (JJP, FA) presented findings to the team during a bimonthly call, which confirmed and nuanced interpretations of the data and furthered critical analysis.

### Ethical approval

The University of California San Francisco Committee on Human Research, the Ethical Review Committee of the Kenya Medical Research Institute, the Makerere University School of Medicine Research and Ethics Committee, and the Uganda National Council of Science and Technology all approved this study (UCSF 20-32144; KEMRI No. 4173; UNCST No. HS1239ES; SOMREC No. 2020-29). All study participants provided written confirmation of informed consent to participate in the study.

## Results: three narrative sub-types

The narratives we present below came from seven participants (3 female, 4 male), ranging in age from 18 to 39. Five narratives are from participants in Kenya and two from Uganda. In comparison to the overall demographics of the qualitative DCP study (37 female, 18 male 18; age range 15 to 58; 30 Kenya, 25 Uganda), men and Kenyans are more represented, and the age range skews slightly younger.

Our participants deployed several identifiable patterns in their illness prevention narratives, each bearing a relation to contemporary African narrative genres. Because local narratives are informed by local genres, and these genres may not entirely match up with Euro-American models, we have chosen to examine these prevention narratives through the lens of three genres as understood through African models: the trickster, the quest, and the tragic hero.

### Trickster narratives

We begin first with the ways in which HIV illness narratives are known and socially deployed in order not only to showcase the shape of a more traditional illness narrative as it is known socially in the East African context, but also to introduce the first narrative type from our cohort. We call this first type a ‘trickster’ narrative, based on the well-known character type from both East and West African folktales (and their continuation in the nineteenth-century African diaspora of the Southeastern US).

### Narrative 1 (Trickster — female)

At first, I duped him that I am HIV positive, and I am on medication (laughter). When he first came across the medications, he was shocked a little and he was worried. Then he asked, ‘Madam I found some medication in your bag. Which drugs are they for?’ Then I was like, ‘Those are my pills.’ Then he said, ‘Which drugs are they?’ I replied, ‘I am on medication.’ Then he was like, ‘How are you on medication?’ I replied, ‘I am sick, that is why I am on medication. They are HIV medications. They prolong life.’ He asked, ‘When did you test for HIV?’ Then I was like, ‘I have been testing for HIV – even when I went to the clinic the other day I was tested for HIV. The result turned positive, so I was given these medications and that is why I am taking them.’ Then he was like, ‘Now, you cannot say even if you are on medication!’ (laughter) I told him, ‘No, I cannot tell the person who brought the infection between me and you. I decided to take medication to prolong my life, as I care for my children. If you would like to know your status, then you are free to do that.’ He said, ‘Now, you want me to go for the test, yet you had been tested long ago – and you are currently taking your medication!’ Then I told him, ‘No problem; I can offer you an HIV test now, when the results turn positive, I can divide my medication with you in case you are still afraid of going to the hospital. When I went back to the hospital, I informed the provider.’ since I was given the self-test kit on that day. When we were about to sleep, he was like, ‘Why don’t you test me then?’ I said, ‘If I test you now and you turn positive, what will I do since it is nighttime, and I also could not call the provider – she’s gone to sleep. Now what would I do if you fainted on me?’ Then he was like, ‘You do not know whether I am sick (infected) or not.’ I replied, ‘You are sick because we are living together, yet I am sick as well and your immunity is so weak. You can end up fainting on me.’ He was like, ‘Just test me; we have talked about it.’ When I tested him and the result came back, I disclosed to him that I am HIV negative. Then he said, ‘It is okay.’ — *39 y.o. female, Magunga; OPD Kenya*

In this first example, an existing sickness narrative form is cleverly co-opted by an individual taking illness-prevention medication to buy social space and excuse her prevention from closer scrutiny while tricking her partner into finding out his status. In animal stories from the region, the narrator is taking the classic role of Hare, who tricks larger animals of prey such as Lion and Hyena, to escape from difficult situations. (Some may be familiar with this character as Br’er Rabbit, from *Uncle Remus’ Tales*, which continue this genre within the African diaspora of the nineteenth-century United States (Harris 1955).) The participant employed a known sick role script: “I’m positive, so I’m on medication, but I don’t know where I acquired the infection. You should test, too.” This indicates that HIV treatment is well enough known that it can be used as an excuse or ploy to explain a situation to someone not in the know. Like in the folktales, by the end of the story the truth is revealed. Knowing he was hoodwinked by a clever partner, rather than get angry, the man simply says, ‘It is okay.’

The second narrative of this type illustrates how for the narrator, being a trickster is part of the narrator’s charm and savviness. He deploys that savviness twice, in two separate incidents, showing how this is part of his character:

### Narrative 2 (Trickster — male)

I: Which of them were options that you haven’t been offered before?

P: Okay, I had never used any of those methods in the past. I have only used them once when I got exposed to HIV. Therefore, I was enrolled on PEP only by the SEARCH staff.

I: Please share with me about the risk you were exposed to?

P: Well, … (chuckles) … people always fall at risk; I had a girlfriend who fell in love with me, and I didn’t know what happened. Therefore, during the holiday she visited me, and we had sex. Then later, when I was trying to dig deep into her history, I heard she was infected and I was not sure about that since I was new in that area. I could not know the truth; it could be true or not. When I gathered courage and asked the girl indirectly – since some can be demoralized if you ask them directly – I tried to bring her closer just to understand about her situation and she was not sure of herself. Thereafter, I thought it wise to seek care since I am the one who will suffer at the end of it all. I may waste my time pressuring the lady, yet I will not get help from her; so I went to the hospital to seek help. It was during the holiday and when I went to the hospital, I was helped. … *Later* … Actually, when I arrived at the hospital, I was like, ‘No, let me go back home I am not sick.’ Then something just encouraged me from within myself, an innate propellant. When I reflected about the kind of life I would like to live, and the things I would like to do in my life, I gathered courage and went to the doctor. I have also witnessed my friends dying of HIV/AIDS. I know quite a number of them. I told you earlier that I am a celeb who usually socializes very much with friends. I also heard that my parents succumbed to HIV, but I do not know the naked truth about that. Therefore, I was like, ‘I want to go far and to go far, first I have to be healthy and I do not need to ignore anything.’ Then I decided to go in and when I reached the doctor’s desk, first I inquired with them if they were providing HIV testing services. Then she asked me, ‘Why do you want to test for HIV?’ Then our discussion started from that point; it was like my turning point, though I knew from the get-go that even if I am infected, it would not come out positive at that particular time. At first, I lied to them that I cut myself with a certain knife which was used by another person. Then they were like, ‘Which knife?’ As they continued to dig deep about me, I was also opening up slowly by slowly because the discussion was heading to my direction as per my expectation. Thereafter, I disclosed to them about what happened and they really helped me. Even now I am telling them that they really helped me a lot. — *18 y.o. male, Magunga; OPD Kenya*

Like the first example, this too is a type of trickster narrative, with several examples presenting an image of the narrator as charming, as when he sought information about his girlfriend’s status, or clever, as when he led the providers into a space where he was able to open up to them. It also has a moral derived from reflection on his parents and friends who passed on, and how he wanted to take their gift of life to him and turn it into something, desiring to go far in life by ensuring his health. Thus, a precipitating event plus a narrative of friends who had passed away and reflection on his own risk level led the participant to seek care. All of this is framed in tropes drawn from both trickster and, as we explore next, contemporary Swahili quest narratives.

### Quest narratives

The ‘new’ Swahili novels often involve quest narratives. A feature of these Swahili-language quests is an ordinary protagonist who looks around or learns the source of the problems in their dystopian world. Often the protagonist encounters a guide, seeks to uncover the truth of a situation, and in the end awakes from a cautionary dream with the realization that if we do not change, the nightmare vision the hero saw will come to pass (Gromov 2019). While none of our narratives illustrates the full complement of events in a quest narrative, three of our prevention narratives do illustrate portions of these quest tales.

### Narrative 3 (Quest — advice)

Most of my friends are older than me; they guide me on how teenage life is. Therefore, one of them married a lady who was HIV-positive, but he did not know. Initially, they were using condoms until they went for the test and they found out that the lady was HIV-positive and he was HIV-negative. So the provider advised him to take PrEP then he enrolled to it. He is the one who told me how effective the drug is. — *19 y.o. male, Oyani; OPD Kenya*

In a quest narrative, the hero is often given a gift or wise counsel early in the story. The hero can either prudently adopt the advice or ignore it. In this example, the participant’s friend related an illness-prevention narrative to the participant, which the participant found convincing. This provided the exigency whereby he decided to prevent the illness by following a peer example, taking the advice he was offered. Sometimes, though, counsel comes in the form of dire warnings, which can appear almost as folktales within quest narratives, like the following account:

### Narrative 4 (Quest — warning)

You may not really tell without observing the true scene, but I heard about it from other people; I was still schooling. She was my classmate, and she was the one who told us that the condom remained in her private part but when she was taken to the hospital, the condom did not come out. She was supposed to go for an operation. Unfortunately, there was no money hence she passed because of that – though I haven’t much information on how the scene went. — *33 y.o. female, Sena; ANC Kenya*

This narrative is almost more like a folktale warning people of consequences and interestingly shows why someone chose the biomedical option of PrEP over an external material barrier like condoms. The account is brief but advises the audience to make alternative choices. It relies on the social fact of poverty as a reason for not being able to access medical care. The everyday experience of poverty is plausible enough to overcome other more implausible elements of the story, lending it an emotional appeal of credibility, even if belief is taken with a grain of salt. More of a counter-narrative in that it relates the dangers of a certain illness prevention method, it follows a similar same pattern of peer example above: “my friend said” is followed by relating “this negative event happened which can happen to me.” Together, the two lead to the logical conclusion, “therefore it’s not the prevention method for me in my search to prevent HIV.” It is useful for highlighting how a prevention narrative falls short: poverty imposes risks that medical options cannot prevent.

The next narrative illustrates the interplay of providence-fate and personal responsibility, which also often feature in the hero’s call to the quest.

### Narrative 5 (Quest — failure to heed advice)

I: Why have you not opted for PEP after any exposure ever since you enrolled in the study?

P: It was my fault not to access PEP the last time I got exposed. I called J (the study clinician) after 72 hours had elapsed. He just urged me not to repeat the mistake and to make sure I call in good time. …I felt so exposed because it was my first time meeting the girl and at the time, we were consuming alcohol. I got attracted to her and suggested that we spend the night together in a hotel room which she agreed to. We then had unprotected sex because we were all tipsy, accompanied by high libido at the time. I then concluded that if I will be safe after this, it is by God’s plan; but if I get infected, it will be out of my lust and I will have myself to blame. — *24 y.o. male, Sibuoche; VHT Kenya*

Quest narratives often embed cosmologies in their accounts, referencing entities like djinn and local spirits, or more ‘diffuse’ concepts like divine care and grace. In this case, the narrator recognizes the potential consequences of a past event and knowledge of what to do next, yet nonetheless fails to act in time. Like the first failure in a quest, the hero approaches a guide (the study clinician) who comforts him, and the narrator subsequently affirms that in the future he intends to act differently. Similar to some of the ‘new’ Swahili novels, in which nightmare and indecision teach the protagonists that if they do not change the unwanted consequences will follow (Gromov 2019). Here, the participant adopted a more optimistic providential-responsible worldview reflecting the theme of desire versus fate, in which whatever consequences might have happened were due to either divine goodwill or personal irresponsibility — with the consequent follow-through that it is the participant’s responsibility in the future to request PEP within the window period.

### Luanda Magere and *tragic hero* narratives

Tragic heroes are well known throughout African literature and orature, ranging from folk heroes like Luanda Magere to the main characters of contemporary novels. The tragedy of these heroes can stem from inherent flaws or from the discovery of the secret sources of their power. The folktale of Luanda Magere, for example, tells of a Luo warrior of incredible strength and skin hard as stone, who could not be defeated by the neighboring Kalenjin tribe, at that time enemies of the Luo. The Kalenjin decided to use subterfuge to discover his weakness and sent a woman to Luanda Magere, who took her as a second wife. Eventually, when the first wife was away, the Kalenjin wife had to administer medicine to him and in the process accidentally learned the secret of his great strength, which lay in his shadow. Letting out the secret of his strength led to his downfall: The second wife returned to her tribe and told them how Luanda Magere could be defeated. The tribe then attacked, but Luanda Magere pushed the Kalenjin warriors back until one warrior recalled that it was Luanda Magere’s shadow which gave him power. Throwing a spear at the shadow, the warrior vanquished Luanda Magere (Miruka 2001; Nzuki 1972; Selman and Battye 2016).

We have two narratives in which someone discovers the narrator was taking PrEP, a secret that the narrator had hidden from others in the household. In the first narrative, a husband is confronted by his wife:

### Narrative 6 (Luanda Magere)

I: So what services have you been receiving from this hospital?

R: When I started with this program, I got medication and as I was told in the beginning, one cannot be infected with HIV when they are exposed if they have been taking it. I asked if it had any effect on the body, and so I got interested and went with it and started swallowing. I have a wife, but I kept the medication a secret. She at times works away from home so when she is not there, this medication helps me a lot in protection.

I: What about when she returned?

R: Before I stopped taking it, she came to find out about the medication, and I took some time and explained to her. She asked why I was taking it, when she was around? I said the time she spends away, I may meet someone and I cannot avoid such so I need to protect myself. She first said I was infected with HIV and never disclosed. We had a heated discussion, and I got the results and I showed her. Since she knows how to read, she calmed down. So I talked with the doctor if I could pause a bit since my wife was around and there was no high risk. What was causing the risk is that my wife was working from far away. — *32 y.o. male, Itojo; OPD Uganda*

This first example alludes to PrEP as a ‘boon’ gifted to the hero. The narrator decides to use the medication, knowing his own fondness for women when his wife is not around. The wife discovers the medication and being at home, convinces him that it is not necessary to take. The tale is open-ended, in that it does not end with a ‘downfall,’ but rather in the resolution of a rupture between husband and wife once the husband decided to stop taking PrEP. In this respect, the narrative does not quite parallel the story of Luanda Magere, which does end in tragedy after the discovery of his secret.

Apart from the classical folk tales of heroes, the contemporary novel form of the ‘tragic hero’ in African literature often tells the story of someone caught between upholding tradition while the community around them opts to embrace change or alter those traditions. Achebe’s *Things Fall Apart* and Ngũgĩ’s *A River Between* are characteristic of this conflict (Achebe 1994; Ngũgĩ 2015). Our final illness prevention narrative strikingly parallels this dynamic. The narrative begins with a prompt asking the participant what her initial impressions of the study were.

### Narrative 7 *(tragic hero)*

I was impressed with the fact that the pills could prevent me from HIV infection because in my life, I really fear being sick. For instance, I always feel bad even when I am suffering from runny nose. I fear HIV as well because I have seen many people who are infected with HIV the way their lives are; they are really suffering and most of the time I am like, “Ooh God what would I do in case I got infected, I really fear this virus.” Then I thought this program would be of great help to me, and that is why I enrolled to the study. — *22 y.o. female, Sena; ANC Kenya*

However, her secret was discovered, and she did not continue PrEP, so hers is a prevention narrative, interrupted; and this is where the conflict of the narrative becomes clear:

P: I took PrEP for a week.

I: Thereafter your husband got the medication?

P: Yes, I put the medication inside my box where my clothes are, unfortunately he found them. I did not know what he was looking for inside there… No – he was not the one who got them at first, but his sister (my sister-in-law). Then she took them to her mother (my mother-in-law) and my sister-in-law approached her mother like “*N*— is taking ARVs!” I was called, sat down, then I told them, ‘Those are not ARVs. Instead, it is ongoing research being conducted about PrEP, which is a prevention medication.’ We quarrelled for some time, then I was like, ‘This is a research study that I joined, and you cannot force me to leave or stop taking part because it is my own decision.’ I knew these are HIV prevention drugs.

I: How did your mother-in-law react when her daughter took these drugs to her?

P: I suspect my mother-in-law was also taking these drugs because she was like, ‘*N*—, you have started taking these pills, is K (participant’s husband) aware about that?’ Then I replied, ‘No, I have not informed him, but these pills are not ARVs; instead they are PrEP, which I am taking to prevent me from HIV infection because I may not know how K is moving around and he does not know how I may be moving as well. Therefore, I am taking them to protect myself.’ Then she said, ‘Never again take those pills!’ Then they informed K (my husband) who also refused that I should not take those pills again.

I: Why do you think your mother-in-law was against your PrEP use?

P: I do not know why she refused because I even asked her the other day, ‘Why did you say I should not take those drugs?’ Then she was like, ‘K— (participant’s husband) cannot cheat on you. K— loves you.’ Then I replied, ‘You may not know.’ Then she said, ‘Do not take those medications again!’ — *22 y.o. female, Sena; ANC Kenya*

The complex story of this patient conforms rather remarkably to the tragic hero novels, in which the protagonist is caught between the old traditions of filial piety and the new technology of PrEP. Like in the Swahili novels, the narrator begins from a situation of risk and uncertainty; she feared seroconversion and she recognized her risk. When presented with the opportunity to take a prevention option, she accepted. However, after the medication was found, and despite pushing back against her mother-in-law and husband, who adhere to older traditions, she eventually gave up the prevention option. The narrator could both foresee the tragedy that will come about if she doesn’t take PrEP, and the tragedy that will happen if she does. In contrast to other tragic heroes in African literature, the regenerative component here is not clear. The example thus delimits what an illness prevention narrative can do when socially deployed — and points the way for an intervention which invokes community consensus, rather than individual choice as the solution. In other words, the outstanding regenerative component must be performed by the community to create a more secure future world.

## Discussion

Preventive care is embedded within social and cultural relations while also falling within the institutional purview of both clinicians and public health officials. As such, prevention narratives are worth studying for their own sake, as well as for what they can tell us about suffering and society more generally. In this paper, we presented seven narratives concerned with biomedical illness prevention, gathered through semi-structured, in-depth interviews during a dynamic choice HIV prevention intervention study. We presented these accounts to complicate the notion of an illness narrative by focusing on narratives about preventive care while connecting them to contextual African narrative genres. Based on our analysis, we argue that illness prevention narratives can indeed exist as a subset of illness narratives.

The narratives describe social roles and relationships and how they influence people navigating prevention. Like the stories of tragic heroes in African literature, several illness prevention narratives revealed disruptive events beyond the potentially unsettling event of potential infection caused by a ‘close call’ or high-risk exposure. These disruptive events highlighted the conflicts that a biomedical prevention option can produce between unquestioned traditions and accepting changing possibilities in community life. These conflicts included heated discussions with spouses and romantic partners, which at times extended through the household to include in-laws of various sorts; women and generational differences in particular were at the center of these stories as both protagonists and antagonists. Disruption was also sometimes caused when taking biomedical prevention methods were discovered after a period of secrecy; but that secrecy itself brings into focus the fact that healthcare decision-making in these settings is both inter-generational and individual-communal in nature.

The tragic genre is recognized in that infection and prevention efforts can equally affect the relationships and people involved in these narratives. Even in the face of a communal decision to change attitudes towards married people taking PrEP, social mimesis — following social expectations through imitating the behavior of others — could still lead to tragic conflicts between those individually upholding tradition in the face of whole communities who collectively advocate for social change, as shown in classics of African literature.

Illness narratives give voice to suffering (Kleinman 2020). Our prevention narratives express suffering by articulating memories of a setting in which AIDS was left untreated as well as in the tragedy of being caught between the norms of filial piety, marital devotion, and biomedical technologies. Social suffering refers to the transpersonal and familial engagement with pain, cultural models for moral experiences, and “what political, economic, and institutional power does to people and, reciprocally, … how these forms of power themselves influence responses to social problems” (Kleinman, Das, and Lock 1997; Kleinman 1997). If social suffering is obvious in the tragic figures who foresee the results of their actions as they are caught between tradition and communal adoption of new forms of living, it is also known in the quest narrative forms as well. In these illness prevention narratives, the suffering concerns avoiding not only a subjective, private experience but also relates to suffering as seen through outside experience in a community of people who have died from AIDS. Thus, certain prevention narratives bear witness to growing up, maturing, or making one’s way through communal memories of collective suffering, though the narrators also give voice to a sense of agency and opportunity in harnessing a biomedical technology to evade further suffering (Kleinman 2000). In this respect, certain illness prevention narratives reflect the quest narratives of the ‘new’ Swahili novels, in which the protagonist must navigate the use of certain technologies over others in order to rectify the problems of the present and create the future world, sometimes through a direct encounter with the social meaning(s) of their chosen prevention method (Gromov 2019; Frank 2009; Ezzy 2000; Wilkinson 2004).

In this respect, illness prevention narratives, like illness narratives more generally, can be oriented towards time or outwards to others (Ezzy 2000). In particular, prevention narratives are by their nature future-oriented, or in an African context, oriented towards the presence and consciousness of the yet-to-be-born and the honour due to lessons imparted by the still-present ancestors. The most obvious example from our cohort is the celebrity participant (Narrative 2), who consciously reflected on what sort of life he wanted to have in the future. Notably, this reflection was inclusive of his ancestors by blood and friendship, following already noted patterns of African consciousness of time. From a Western or Northern perspective, and in contrast to other illness narratives, prevention narratives focus on *preventing change* over time, whereas illness narratives center the actual change that an illness brings about (Hydén 1997). In both cases, however, the narrative includes efforts to construct some continuity through an illness or potential illness (Kelly and Dickinson 1997). In the context of our East African narratives, that continuity is linked with those who have died as well as the descendants for whom one is responsible. Intriguingly, the trickster narratives are particularly adept at bringing out these links, as with the participant who in deceiving her partner about her HIV status, presented a justification touching on her children (Narrative 1). This justification invoked an acceptable moral worldview.

Our narratives also illustrate other features of African literary genres, such as protagonists with flaws which can be affectionately laughed at, though they may have serious consequences. Most prevention narratives in our cohort are, in fact, ‘comic’ plots (in the Aristotelian sense) because with few exceptions, the person returns to life as before, not necessarily deeply affected by the experience. Yet this, too reflects not only the point of taking up prevention methods (moving from risk to security), but also the holistic attitude within African literature towards the reversibility of fortune and fate.

### What moral purpose do illness prevention narratives serve?

Morality orients people to others and to their own values in relation to the world. ‘Moral narratives’ provide accounts of, and help to constitute, changes between the person, the illness, and social identity. This type of narrative helps to (re-)establish the individual’s moral status, constituting the moral self, or helps maintain social distance (Riessman 1990). In our prevention narratives, both the celebrity and the savvy girlfriend are concerned with ‘impression management’ in relating to others (Narratives 2 and 1). In these narratives, the relationship is twofold: first to the interviewer, and second to the persons included in the narrative. Thus, the accounts morally position the narrators as clever tricksters who manage not only to protect themselves through PrEP to be there for their children or take account of their deceased parents and friends, but also to get a partner to test for HIV or ease oneself into discussing the actual situation with providers. In this respect, both narrators benefit from the telling of the narrative (Shapiro 2011). Still other narratives conform to morality plays and conversion narratives of learning a lesson through a close call (Narrative 5) (Hawkins 1990; Shapiro 2011).

Further, while prevention can be considered ‘restitution’ to the community in the sense of embracing responsibility (Frank 2013), responsibility to self and responsibility to community can come into conflict.

In an African context of navigating neo-colonial social change, the mother-in-law narrative in which filial piety and self-responsibility are placed in tension also advances a moral agenda, showing the narrator’s value priorities in relation to others (Narrative 7). That narrative demonstrates why moral purpose is centrally important for public health officials to account for, especially when prevention becomes socially and politically polarized between those who hold to older traditions against the consensus of the community in considering changes.

### Therapeutic value and applications

Therapy aims to restore proper functioning in the world. For prevention narratives, the goal may not be transformation, but rather contextualizing a problem — HIV prevention, in our case — by using familiar tropes or genres to understand it in ways that make the problem easier to handle.

Illness prevention narratives help clinicians and providers co-create a therapeutic agenda with people facing challenges in HIV prevention by objectifying those challenges in ways familiar from the stories people encounter within a particular cultural context. Instead of the problem being intangible or inherently tied to a person, it is understood by drawing parallels between an analogous story and the concrete elements in a personal situation. The result is a paradoxical distancing from the problem while making a known story pertinent to the narrator’s immediate needs.

These stories are relevant to public health because prevention narratives highlight how HIV prevention methods affect specific relationships: romantic, familial, and communal. While challenges in dyadic relationships may be overcome by learning from the folklore of hare and hyena (Narratives 1 and 2), other dynamic relationships call for marshalling different resources. Just as the tragic novels illustrate the need for communal consensus, so also in combination with Narrative 7, we can see how public health approaches to HIV prevention must include community-wide dialogue and consensus, rather than be approached as simply an individual matter. Otherwise, family contexts pressure individuals to make tradeoffs between moral priorities in their fashioning of the self and HIV-prevention efforts. Yet the limits of such approaches are found in the Swahili novels, which like Narratives 3 and 4, hint at how people navigate neo-colonial contexts in which communal consensus can be distorted by corruption, age, or misinformation. Thus, while moral priorities are communally imparted, they are individually realized through both stories and experience.

### Theoretical directions

Finally, the inclusion of illness prevention narratives can further theory in several key areas. Initially, illness narrative theory focused on the constitution of the self in a social context more often characteristic of individualistic rather than communal cultures. Narrators experienced the tension of giving public voice to private experience, a tension recognized three decades earlier by Hannah Arendt with respect to pain and politics (Frank 1994; Arendt 2013). Yet giving public voice to private experience is a social media norm today. Because prevention relies partly on the vicarious experience of an illness rather than personal experience, however, prevention narratives pivot from purely subjective experience to the experience of society and community context. Illness prevention narratives thus shed light on those social processes and social ties which biomedical prevention options have the potential to either transform or re-inscribe as certain social norms. Illness prevention narratives thus allow us to probe other social roles associated with illness and care of the self, beyond that of the sick role, therapeutic citizenship, or participation in a moral health crusade (Jurecic 2012).

Another narrative studies question that illness prevention theories raise in this context is whether prevention can be a metaphor. While several historical works bear out the relation between the ‘cleansing of society’ through persecution or segregation of people deemed to represent ‘moral ills,’ the idea of biomedical prevention being a metaphor is still rather new (Hydén 1997; Brown 1990; Moore 2008; Foucoult 1975). Moreover, prevention does not seem to feature heavily in literary works — at least not yet (Sontag 2001). How biomedical prevention, moral contagion, and metaphor relate is an open field for study, and while we have some evidence from our population that using PrEP is considered more ‘morally dangerous‘ than condoms, we do not have fully fleshed out narratives to this effect, nor did we clearly see PrEP used metaphorically.

### Limitations

Narratives should not be fragmented into codes, but taken whole, as in an enclave of an interview (Riessman 1990). Although the excerpts are not as long nor as coherent as what would be obtained by asking someone with a diagnosed disease to “tell me the story of your illness,” they are sufficiently complete to provide a representative feel of the types of illness prevention narratives we encountered and how they relate to African literary genres. Further, while we cannot ascertain from our results how central these stories become to a participant’s life, their immediate deployment in the interview created a self in several instances: the celebrity, the teenager, the savvy girlfriend, the dutiful daughter-in-law. Finally, our narratives are localized within the African setting; this may be a limitation for how other settings with similar prevention-uptake challenges could adapt our findings because greater abstraction might be necessary.

## Conclusions: why study a prevention narrative?

Our paper contributes to the literature by looking at illness prevention and by bringing contemporary African literary theory to bear on the examination of narratives from an African context. Illness prevention narratives give public health researchers scripts which resonate with the moral and cultural sensibilities of a given population. As snapshots into cultural life, highlighting tension between society and self-presentation (Bury 2001), they provide the perfect opportunity for public health communicators to shape public perception and smooth the way for people to embrace their own agency in ways which build communal consensus around technological and social change.

With the introduction of HIV prevention options like PrEP, the uptake of cervical and anal cancer prevention through HPV vaccines, and the increased visibility of anti-vaccination groups, we argue it is time for public health officials to discuss the narratives and counter-narratives which concern not only risk but the prevention and elimination of suffering and disease. This is even more urgent as these narratives are not only taken up individually but are also deployed politically. Because of this, both illness narratives and *illness prevention* narratives should be attractive subjects for analysis and deployment by the medical social sciences, a central concern of which is the public health.

## Supplementary Material

Supplementary Material

## Data Availability

The data from which the narratives of this paper are drawn are available on request from the corresponding author, but due to ethical restrictions around confidentiality of the study participants are not publicly available.
