# Supplementary material for: Illness Narratives Without the Illness: Biomedical HIV Prevention Narratives from East Africa
Source: J Med Humanit. 2024 Jun 26;45(4):345–68. doi: 10.1007/s10912-024-09862-0 (PMC11578797; doi:10.1007/s10912-024-09862-0)
Supplement: Supplementary file 1 — Supplementary file1 (DOCX 22 KB) [file 10912_2024_9862_MOESM1_ESM.docx]

| **Potential Narratives: Green background items were not used in the manuscript** |
| --- |
| “At first, I duped him that I am HIV positive, and I am on medication (laughter). When he first came across the medications, he was shocked a little and he was worried. Then he asked, ‘Madam I found some medication in your bag. Which drugs are they for?’ Then I was like, ‘Those are my pills.’ Then he said, ‘Which drugs are they?’ I replied, ‘I am on medication.’ Then he was like, ‘How are you on medication?’ I replied, ‘I am sick, that is why I am on medication. They are HIV medication. They prolong life.’ He asked, ‘When did you test for HIV?’ Then I was like, ‘I have been testing for HIV – even when I went to the clinic the other day I was tested for HIV. The result turned positive, so I was given these medications and that is why I am taking them.’ Then he was like, ‘Now, you cannot say even if you are on medication!’ (laughter) I told him, ‘No, I cannot tell the person who brought the infection between me and you. I decided to take medication to prolong my life, as I care for my children. If you would like to know your status, then you are free to do that.’ He said, ‘Now, you want me to go for the test, yet you had been tested long ago – and you are currently taking your medication!’ Then I told him, ‘No problem; I can offer you an HIV test now, when the results turn positive, I can divide for you my medication in case you are still afraid of going to the hospital. When I go back to the hospital, I would inform the provider.’ It found when I was given the self-test kit. When we were about to sleep, he was like, ‘Why don’t you test me then?’ I said, ‘If I test you now and you turn positive, what will I do since it is night time, and I also could not call the provider – she’s gone to sleep. Now what would I do if you fainted on me?’ Then he was like, ‘You do not know whether I am sick (infected) or not.’ I replied, ‘You are sick because we are living together, yet I am sick as well and your immunity is so weak. You can end up fainting on me.’ He was like, ‘Just test me; we have talked about it.’ When I tested him and the result came back, I disclosed to him that I am HIV negative. Then he said, ‘It is okay.’” [OPD Kenya] |
| “*I: So what services have you been receiving from this hospital?* R: When I started with this program, I got medication and as I was told in the beginning, one cannot be infected with HIV when they are exposed if they have been taking it. I asked if it had any effect on the body, and so I got interested and went with it and started swallowing. I have a wife, but I kept the medication a secret. She at times works away from home so when she is not there, this medication helps me a lot in protection. *I: What about when she returned?* R: Before I stopped taking it, she came to find out about the medication, and I took some time and explained to her. She asked why I was taking it, when she was around? I said the time she spends away, I may meet someone and I cannot avoid such so I need to protect myself. She first said I was infected with HIV and never disclosed. We had a heated discussion, and I got the results and I showed her. Since she knows how to read, she calmed down. So I talked with the doctor if I could pause a bit since my wife was around and there was no high risk. What was causing the risk is that my wife was working from far away. *I: Where does she work from?* R: She works in Fort portal. So during COVID, she would be home. It’s not that I refused the medication – because the services were of great importance [OPD Uganda] |
| “For instance, there was a day I took my pill and then I went and had sex with a certain lady; unfortunately, she was HIV positive. When I later tested after three months, I learnt that I was still okay (HIV negative). I am now sure that this pill works, and I would like to continue taking it.” [OPD Kenya] |
| “*I: Why have you not opted for PEP after any exposure ever since you enrolled in the study?* P: It was my fault not to access PEP the last time I got exposed. I called J (the study clinician) after 72 hours had elapsed. He just urged me not to repeat the mistake and to make sure I call in good time. …I felt so exposed because it was my first time meeting the girl and at the time, we were consuming alcohol. I got attracted to her and suggested that we spend the night together in a hotel room which she agreed to. We then had unprotected sex because we were all tipsy, accompanied by high sex urge at the time. I then concluded that if I will be safe after this, it is by God’s plan; but if I get infected, it will be out of my lust and I will have myself to blame.  *I: Did she even suggest condom use before the sexual act?* P: She did not – I told you we were all tipsy and all of us failed to think in that direction.” [VHT Kenya] |
| “P: In most cases, I get sick of STIs and STDs because of having sexual intercourse with my man – and these would bring me problems because I would use a lot of money for treatment. So I would like to receive those services when I come to pick up my medicine because it is very costly for me.  I*: Do you receive treatment from private health facilities?*  P: I go to clinics so to make it easier for me, which can be provided. Also, other diseases develop that I suffer from because of taking these medicines. And I wondered why, but I came to understand that I could be having my other disease not that it is the effect of the medicine that I take. Therefore, I accepted and said I needed to talk to my health providers so that when they tell me that the medicine is not the cause [of these effects] but rather my diseases, then I can receive the services for these diseases that are within my blood.  *I: What happens to you?* P: I get allergies and the eyes can’t see well.” [OPD Uganda] |
| *“I: How confident were you that it would be effective?* P: Having heard this from my friends, I was confident about it because they had used it and it helped them, so I also decided to use it.  *I: What did your friends tell you about it and what initiated the story?* P: Most of my friends are older than me; they guide me on how teenage life is. Therefore, one of them married a lady who was HIV-positive, but he did not know. Initially, they were using condoms until they went for the test and they found out that the lady was HIV-positive and he was HIV-negative. So the provider advised him to take PrEP then he enrolled to it. He is the one who told me how effective the drug is.” [OPD Kenya] |
| “*I: Which of them were options that you haven’t been offered before?*  P: Okay, I had never used any of those methods in the past. I have only used them once when I got exposed to HIV. Therefore, I was enrolled on PEP only by the SEARCH staff.  *I: Please share with me about the risk you were exposed to?* P: Well, … (chuckles) … people always fall at risk; I had a girlfriend who fell in love with me, and I didn’t know what happened. Therefore, during the holiday she visited me, and we had sex. Then later, when I was trying to dig deep into her history, I heard she was infected and I was not sure about that since I was new in that area. I could not know the truth; it could be true or not. When I gathered courage and asked the girl indirectly – since some can be demoralized if you ask them directly – I tried to bring her closer just to understand about her situation and she was not sure of herself. Thereafter, I thought it wise to seek care since I am the one who will suffer at the end of it all. I may waste my time pressuring the lady, yet I will not get help from her; so I went to the hospital to seek help. It was during the holiday and when I went to the hospital, I was helped. [… Later …] Actually, when I arrived at the hospital, I was like, ‘No, let me go back home I am not sick’. Then something just encouraged me from within myself, an innate propellant. When I reflected about the kind of life I would like to live, and the things I would like to do in my life, I gathered courage and went to the doctor. I have also witnessed my friends dying of HIV/AIDS. I know quite a number of them. I told you earlier that I am a celeb who usually socializes very much with friends. I also heard that my parents succumbed to HIV, but I do not know the naked truth about that. Therefore, I was like, ‘I want to go far and to go far, first I have to be healthy and I do not need to ignore anything.’ Then I decided to go in and when I reached the doctor’s desk, first I inquired with them if they were providing HIV testing services. Then she asked me, ‘Why do you want to test for HIV?’ Then our discussion started from that point; it was like my turning point, though I knew from the time go that even if I am infected, it would not come out positive at that particular time. At first, I lied to them that I cut myself with a certain knife which was used by another person. Then they were like, ‘Which knife?’ As they continued to dig deep about me, I was also opening up slowly by slowly because the discussion was heading to my direction as per my expectation. Thereafter, I disclosed to them about what happened and they really helped me. Even now I am telling them that they really helped me a lot.” [OPD Kenya] |
| *“I: You have mentioned that condoms may affect you sometimes.* P: Yes, it may affect you. *I: Tell me more about that.* P: (Laughs) we had a girl back at home – a condom remained in her vagina. She later died. So I am very afraid of condoms. I do not want to use condoms with fear that they may affect me. When I remember that scenario, I am very afraid.  *I: Please tell me more about that how did condom remain in her private part.* P: You may not really tell without observing the true scene, but I heard about it from other people; I was still schooling. She was my classmate, and she was the one who told us that the condom remained in her private part but when she was taken to the hospital, the condom did not come out. She was supposed to go for an operation. Unfortunately, there was no money hence she passed because of that – though I haven’t much information on how the scene went.” [ANC Kenya] |
| “*I: Okay, what were your impressions of the study at that time?* P: I was impressed with the fact that the pills could prevent me from HIV infection because in my life, I really fear being sick. For instance, I always feel bad even when I am suffering from runny nose. I fear HIV as well because I have seen many people who are infected with HIV the way their lives are; they are really suffering and most of the time I am like, “Ooh God what would I do in case I got infected, I really fear this virus.” Then I thought this program would be of great help to me, and that is why I enrolled to the study.” *I: You have mentioned that you took it for a week.* P: Yes, I took PrEP for a week. *I: Thereafter your husband got the medication?* P: Yes, I put the medication inside my box where my clothes are, unfortunately he found them. I did not know what he was looking for inside there, … No – he was not the one who got them at first, but his sister (my sister-in-law). Then she took them to her mother (my mother-in-law) and my sister-in-law approached her mother like “Nya-Wakula is taking ARVs!” I was called, sat down, then I told them, ‘Those are not ARVs. Instead, it is ongoing research being conducted about PrEP, which is a prevention medication.’ We quarreled for some time, then I was like, ‘This is a research [study] that I joined, and you cannot force me to leave or stop taking part because it is my own decision.’ I knew these are HIV prevention drugs. *I: How did your mother-in-law react when her daughter took these drugs to her?* P: I suspect my mother-in-law was also taking these drugs because she was like, ‘Nya-Wakula, you have started taking these pills, is K (participant's husband) aware about that?’ Then I replied, ‘No, I have not informed him, but these pills are not ARVs; instead they are PrEP, which I am taking to prevent me from HIV infection because I may not know how K is moving around and he does not know how I may be moving as well. Therefore, I am taking them to protect myself.’ Then she said, ‘Never again take those pills!’ Then they informed K (my husband) who also refused that I should not take those pills again. *I: Why do you think your mother-in-law was against your PrEP use?* P: I do not know why she refused because I even asked her the other day, ‘Why did you say I should not take those drugs?’ Then she was like, ‘K (participant’s husband) cannot cheat on you. K loves you.’ Then I replied, ‘You may not know.’ Then she said, ‘Do not take those medications again!’ [ANC Kenya] |
| “*I: Do you think HIV is a bad disease?* R: Yes, according to the way I see people who are suffering from it, it’s a bad disease. *I: What’s their situation?* R:The situation is hard. They become weak and sickly when they miss medication. I started fearing HIV when I was young. We had neighbours who all got sick and died, and at the time – and those would have not have adhered to medication, though nowadays one cannot even know someone is HIV positive; yet they got sick a long time ago. Some got sick in 2013. So that’s why I said I know about HIV.” [VHT Uganda] |
| “*I:* You had mentioned about intimate partner violence and a client you helped. You mentioned referring the client to C to handle it. So how do you feel being able to give such a service? R: There was a time I went to see a participant on their due date. I called the day before to remind them of my visit. The participant wanted to wake up very early and go, and he knew that I was going to delay him. He decided to do the test in the night. At 5am in the morning, I called him and told him that I would be coming. When I got there, he showed me his test strip. I also asked him about the pills he was taking. At that time, he did not have a wife but was cohabiting with a lady I did not know quite well. She was in the bedroom. So when, I left she asked him, ‘We tested for HIV before I came here, now I see this man is asking you about some drugs which I have not seen, what is going on?” She explained to him that we are in a study, and that I am a VHT participating in a research study. Since the woman was not in the study, she assumed that the man was taking ARVs. The woman was not convinced so the man called me and told me that I should pass by his house on my way back. When I got there he told me, ‘This lady would like to ask you a few questions.’ He left me alone with the woman and walked away. I was so confused at that moment until I devised my own way of approaching her. I got out a self-test kit and told her that I would like to test her. Her partner’s kit was also still there, so I put her strip next to his. Her test also turned out to be negative, and that is when she believed that her partner was in a study and there was nothing else. Otherwise she was about to separate with him or it would cause violence.” [VHT Uganda] |
